# 18F-FDG PET/CT-based deep radiomic models for enhancing chemotherapy response prediction in breast cancer

**DOI:** 10.1007/s12032-025-02982-0

**Published:** 2025-08-11

**Authors:** Zirui Jiang, Joshua Low, Colin Huang, Yong Yue, Christopher Njeh, Oluwaseyi Oderinde

**Affiliations:** 1 Advanced Molecular Imaging in Radiotherapy (AdMIRe) Research Lab, School of Health Sciences, College of Health and Human Sciences, Purdue University, West Lafayette, IN 47907 USA; 2 Department of Radiation Oncology, Indiana University School of Medicine, Indianapolis, IN 46202 USA

**Keywords:** PET/CT imaging, Radiomics model, Chemotherapy, Breast cancer, Treatment response

## Abstract

Enhancing the accuracy of tumor response predictions enables the development of tailored therapeutic strategies for patients with breast cancer. In this study, we developed deep radiomic models to enhance the prediction of chemotherapy response after the first treatment cycle. 18F-Fludeoxyglucose PET/CT imaging data and clinical record from 60 breast cancer patients were retrospectively obtained from the Cancer Imaging Archive. PET/CT scans were conducted at three distinct stages of treatment; prior to the initiation of chemotherapy (T1), following the first cycle of chemotherapy (T2), and after the full chemotherapy regimen (T3). The patient’s primary gross tumor volume (GTV) was delineated on PET images using a 40% threshold of the maximum standardized uptake value (SUVmax). Radiomic features were extracted from the GTV based on the PET/CT images. In addition, a squeeze-and-excitation network (SENet) deep learning model was employed to generate additional features from the PET/CT images for combined analysis. A XGBoost machine learning model was developed and compared with the conventional machine learning algorithm [random forest (RF), logistic regression (LR) and support vector machine (SVM)]. The performance of each model was assessed using receiver operating characteristics area under the curve (ROC AUC) analysis, and prediction accuracy in a validation cohort. Model performance was evaluated through fivefold cross-validation on the entire cohort, with data splits stratified by treatment response categories to ensure balanced representation. The AUC values for the machine learning models using only radiomic features were 0.85(XGBoost), 0.76 (RF), 0.80 (LR), and 0.59 (SVM), with XGBoost showing the best performance. After incorporating additional deep learning-derived features from SENet, the AUC values increased to 0.92, 0.88, 0.90, and 0.61, respectively, demonstrating significant improvements in predictive accuracy. Predictions were based on pre-treatment (T1) and post-first-cycle (T2) imaging data, enabling early assessment of chemotherapy response after the initial treatment cycle. Integrating deep learning-derived features significantly enhanced the performance of predictive models for chemotherapy response in breast cancer patients. This study demonstrated the superior predictive capability of the XGBoost model, emphasizing its potential to optimize personalized therapeutic strategies by accurately identifying patients unlikely to respond to chemotherapy after the first treatment cycle.

## Introduction

Breast cancer is the most prevalent and consequential malignancies among women globally, posing a considerable challenge to public health [1, 2]. Although there have been significant strides in treatment approaches, encompassing surgical intervention, radiation therapy, and chemotherapy, yet poor outcomes persist. The early prediction of patient response to treatment constitutes a critical yet unresolved issue [3]. The ability to identify patients unlikely to benefit from chemotherapy after the first treatment cycle has profound implications for oncologic management. It will enable the development of personalized treatment strategies, minimizes patient exposure to ineffective therapies, and optimizes the overall success of treatment regimens.

Advanced imaging techniques, particularly the integration of Positron Emission Tomography/Computed Tomography (PET/CT) with machine learning models, represent a transformative development in oncologic management [4]. This integration harnesses the detailed imaging capabilities of PET/CT alongside the robust analytical power of machine learning to enhance the precision of predictive healthcare models. Recent studies have demonstrated that radiomic features extracted from imaging data can serve as valuable predictors of treatment response in various cancers [4, 5].

The integration of machine learning (ML) into oncology, particularly in the realm of breast cancer, has significantly enhanced diagnostic precision and the prediction of treatment outcomes. Numerous studies have highlighted the effectiveness of ML models in processing complex, multidimensional datasets derived from clinical, imaging, and genetic sources, uncovering patterns and insights often overlooked by traditional analytical methods [4, 6–8]. This advancement has revolutionized cancer diagnosis by enabling more nuanced interpretations of patient data, fostering personalized treatment strategies, optimizing therapeutic efficacy, and reducing adverse effects [4, 6, 9, 10]. Furthermore, ML algorithms have played a pivotal role in risk stratification and prognostic evaluations, advancing personalized oncology by accurately predicting long-term patient outcomes and facilitating tailored clinical decision-making.

Radiomics has emerged as a transformative approach in medical imaging, leveraging advanced machine learning techniques to extract high-dimensional features from medical images, such as those obtained from PET/CT scans [11]. These features, encompassing tumor shape, texture, and intensity, have been extensively utilized in studies aimed at developing predictive models for treatment outcomes in various cancers [11, 12]. For instance, a systematic review and meta-analysis on esophageal cancer highlighted the efficacy of radiomics in predicting survival and treatment response, demonstrating the potential of combining clinical and radiomic features for improved prognostication [13]. Similarly, a study on hepatocellular carcinoma developed a radiomics-based model using multiparametric MRI, achieving enhanced predictive performance with a defined prediction score threshold [14]. These examples underscore the growing role of radiomics in integrating diverse imaging modalities to advance personalized treatment strategies and improve clinical outcomes.

While radiomics has shown immense potential in predictive modeling, its application is not without limitations. One major challenge lies in the manual or semi-automated feature extraction process, which often relies on predefined radiomic features that may not fully capture the complexity and heterogeneity of tumors [15]. Additionally, radiomic models can be sensitive to variations in imaging protocols, reconstruction settings, and segmentation techniques, potentially affecting their reproducibility and generalizability across diverse clinical datasets [16]. To address these limitations, researchers have increasingly turned to deep learning-based approaches that extract deep features directly from medical images.

To address these limitations, researchers have increasingly turned to deep learning-based approaches that extract deep features directly from medical images. Unlike traditional radiomics, which relies on handcrafted features, deep learning enables the automated extraction of high-dimensional, data-driven features that capture the complex and nuanced characteristics of tumors. Recent studies have demonstrated that integrating deep learning-extracted features with traditional radiomics methods can improve predictive accuracy and robustness in treatment response models. For example, Wang et al. developed a multimodality deep learning radiomics model that combined traditional and deep learning features from CT and MRI images to predict pathological complete response in esophageal squamous cell carcinoma, achieving an AUC of 0.868 [17]. Similarly, Wei et al. evaluated breast cancer axillary lymph node metastasis using deep learning radiomics of conventional ultrasound images, finding that the combination of deep learning and traditional radiomics features yielded an AUC of 0.92, outperforming models based solely on traditional radiomics [18]. These findings underscore the ability of deep learning-based approaches to comprehensively capture tumor heterogeneity, thus advancing the precision and reliability of predictive models for personalized oncology care.

While the integration of deep learning-extracted features with traditional radiomics has shown success in other cancers, its application in breast cancer remains underexplored. Few studies have leveraged this combined approach to enhance predictive accuracy in breast cancer, leaving a gap in understanding its potential to improve treatment response predictions. This underscores the need for further research to adapt and validate these methodologies specifically for breast cancer cases. This study aimed to extract FDG PET/CT-based deep radiomic features and use it to develop ML model for predicting chemotherapy response in breast cancer patients. Additionally, this study compared the performance of eXtreme Gradient Boosting (XGBoost), with the traditional ML models such as SVM (Support Vector Machine), Logistic Regression (LR), and Random Forest (RF) to improve the prediction accuracy.

## Materials and methods

### Data collection and imaging preprocessing

This retrospective study collected F-18 Fluorodeoxyglucose (FDG) PET/CT images and clinical notes from 60 breast cancer patients from the Cancer Imaging Archive (QIN-BREAST) [19]. Among these, 29 patients achieved pathological complete response (pCR), while 31 were classified as non-pCR (non-responders or progressive disease). Imaging data were collected at three pivotal treatment stages: prior to treatment initiation (T1), following the first chemotherapy cycle (T2), and after the completion of chemotherapy (T3). For each patient, the average interval between T1 and T2 imaging was approximately 18.9 days (range: 6–28 days), with T2 typically acquired shortly after the first or second chemotherapy cycle. This interval was selected to capture early metabolic changes in tumor response following treatment initiation. All patients had an average interval of 123 days, approximately four months, between the T1 and T3 imaging sessions. In terms of the SUV target volume, the analysis focused on the primary tumor (T). The gross tumor volume (GTV) was delineated on pre-treatment PET images (T1) using a 40% threshold of the maximum standardized uptake value (SUVmax).Then, the GTV on the T1 images was co-registered with T2 and T3 PET/CT images to assess radiological response to treatment. Radiological responses were categorized based on reductions in PET avidity from baseline: a complete response (CR) was defined as a reduction of 75% or more, a partial response (PR) as a reduction between 40 and 74%, and no response (NR) as a reduction of less than 40%. These criteria align with the positron emission tomography response criteria in solid tumors (PERCIST), which standardizes metabolic response assessments in oncology [20]. Implementing such standardized criteria enhances the consistency and accuracy of treatment response evaluations, facilitating better-informed clinical decisions. The imaging data employed in this study were sourced from the QIN-Breast dataset available through The cancer imaging archive (TCIA). This dataset is publicly accessible and has been de-identified in compliance with the Health Insurance Portability and Accountability Act (HIPAA) regulations, ensuring the protection of patient privacy. As the data are anonymized and publicly available, the study was exempt from institutional review board (IRB) approval. Nonetheless, we adhered to all relevant guidelines and regulations governing the use of such data. In accordance with TCIA’s data usage policies, we have cited the dataset appropriately and have not attempted to re-identify any individual participants.

### Software, data extraction, and machine learning models

For this study, Python version 3.8 was used for code execution, model development, and debugging. Radiomic features were extracted from PET/CT images using Pyradiomics. Three machine learning models were implemented: XGBoost, RF, LR, and SVM, within the Python environment for a comprehensive assessment of results. All were conducted within the Python environment to facilitate a comprehensive assessment of the results.

### Radiomic feature extraction

A comprehensive set of 314 radiomic features was extracted from each patient’s primary tumor volume (Gross Tumor Volume, GTV) to facilitate the construction of machine learning models. Specifically, 88 features were derived from the patients’ CT imaging data, while the remaining 226 features originated from the PET imaging data. In addition to the radiomic features, a semi-quantitative analysis of PET parameters and clinical features, including the intervals between the three specified time points, was performed.

**Fig. 1:**
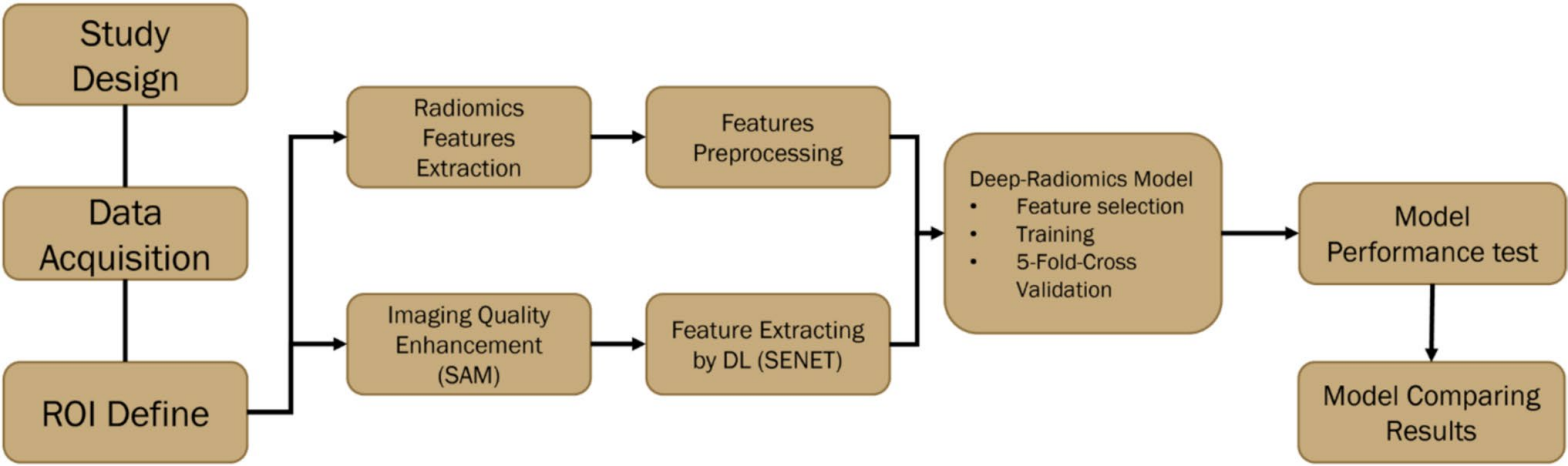
The workflow of deep radiomics models

To enhance the efficiency of the machine learning model computations and optimize the feature weights, all features were subjected to normalization. The performance of each machine learning model in predicting patient responses was assessed using the AUC analysis within a validation cohort. This approach ensured a robust evaluation of the predictive capabilities of the models. The machine learning model workflow is showed in Fig. 1.

### Deep feature extraction

A deep learning model was also developed using the segment-anything model (SAM), specifically employing the SAM_VIT_B_01EC64.pth to enhance the quality of the ROI in the imaging. Additionally, the SENet deep learning model (from SENet.PyTorch) was used to extract additional features from the GTV within the PET/CT images. Following feature extraction, the SelectKBest package was employed to identify the top 200 most significant features based on their predictive power. Deep learning-derived features were subsequently integrated with the original set of radiomic features to form a comprehensive dataset.

### Feature selection and validation

To facilitate a rigorous evaluation of model performance, two distinct datasets were constructed: one comprising only the radiomic features and the other combining both the deep learning-derived features and the radiomic features. These datasets were used for training and evaluating multiple machine learning models, allowing for a comparative analysis of the impact of incorporating deep learning-derived features on model accuracy.

The predictive models were trained and validated using imaging data acquired at two critical timepoints: baseline (T1, pre-treatment) and post-first-cycle (T2). This design aligns with the clinical goal of early response prediction, allowing timely therapeutic adjustments before the second chemotherapy cycle.

In this segment, patients were randomly allocated into training and testing sets, with a fivefold cross-validation employed to ensure a reliable estimation of model performance. The training set was utilized for the training and refinement of models, while the testing set, isolated from the model fitting process, was used to evaluate the performance of the trained models.

## Results

### AUC values and heat map

**Fig. 2:**
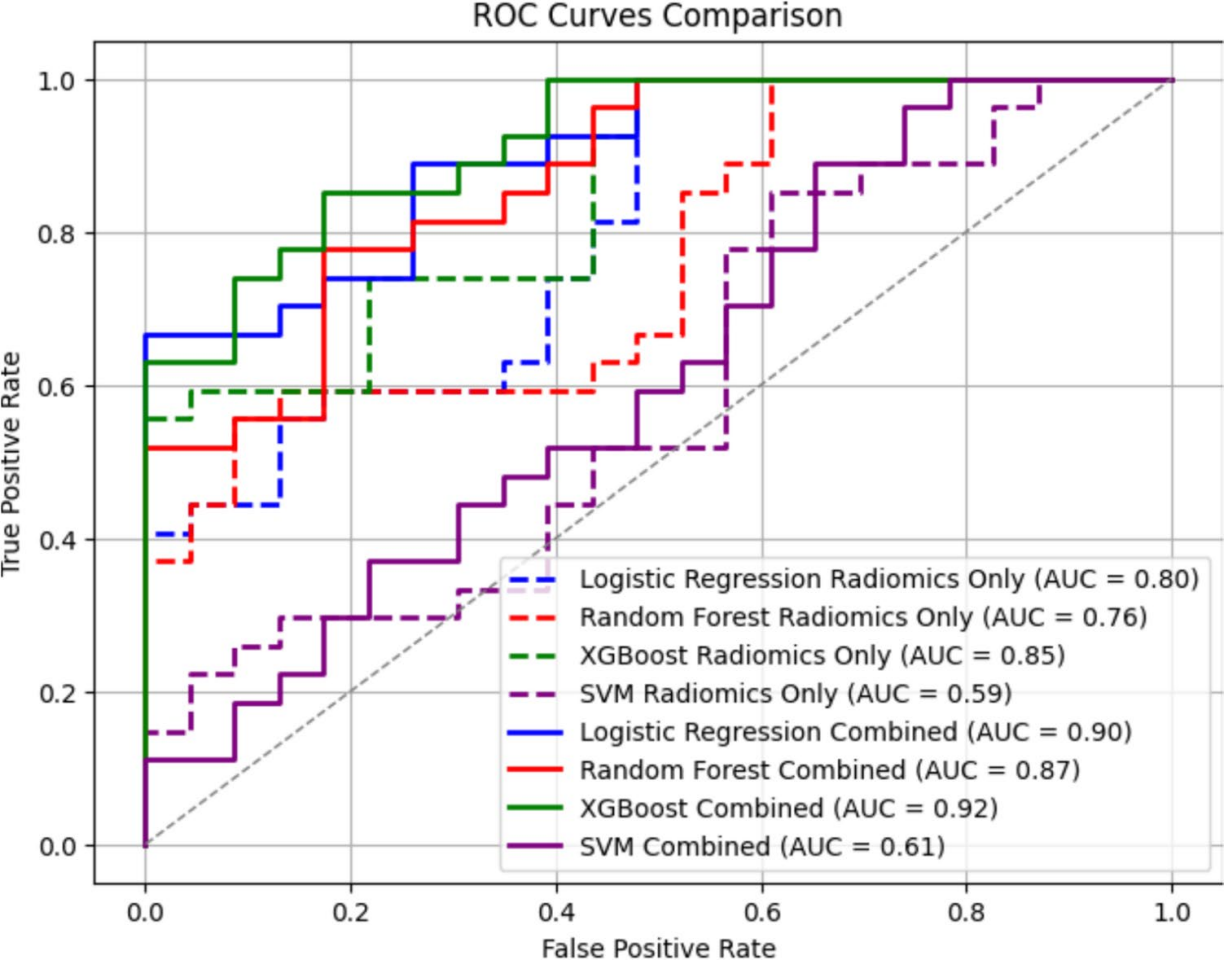
Comparison of radiomics-only and combined model performance using ROC curves

When using only radiomic features, the AUC values achieved by the RF, XGBoost, LR, and SVM models were 0.85(95%CI 0.73–0.97), 0.76 (95%CI 0.61–0.91), 0.80 (95%CI 0.66–0.94), and 0.59 (95%CI 0.41–0.77), respectively, shown in Fig. 2. Notably, only the SVM model failed to exceed the clinical threshold of 0.70, while the other models demonstrated strong predictive performance, with XGBoost achieving the highest AUC value of 0.84, highlighting its superior capability.

After incorporating deep learning-extracted features with the radiomic features, the AUC values for XGBoost, LR, RF, and SVM increased to 0.92 (95%CI 0.847–1.000), 0.90 (95%CI 0.818–0.982), 0.88 (95%CI 0.791–0.969), and 0.61 (95%CI 0.467–0.753), respectively, as shown in Fig. 2. While the performance of the SVM model remained suboptimal, the AUC values for the other models demonstrated significant improvements, underscoring the value of integrating deep learning-derived features with radiomic features.

Statistical significance of AUC differences between models was assessed using DeLong’s test in Table 1. After Bonferroni correction for multiple comparisons (*α* = 0.05/6 pairwise comparisons), the improvement in XGBoost’s AUC from 0.85 (radiomics-only) to 0.92 (radiomics + deep features) was statistically significant (*p* = 0.003). Similarly, LR and RF showed significant improvements (*p* = 0.012 and *p* = 0.021, respectively), while SVM’s performance remained unchanged (*p* = 0.42).

**Table 1:** Performance comparison of radiomics-only and combined models using ROC analysis

	Radiomics only (AUC)	Combined (AUC)	DeLong’s test (*p*-value)
XGBoost	0.85	0.92	0.003*
Logistic regression	0.80	0.90	0.012*
Random forest	0.76	0.87	0.021*
SVM	0.59	0.61	0.42

*AUC* area under the curve

*Indicates statistical significance (*p* < 0.05)

The combined models were further evaluated based on their sensitivity, specificity, positive predictive value (PPV), and negative predictive value (NPV), as summarized in Table 2. Among all models, XGBoost exhibited the most favorable balance between sensitivity and specificity, accompanied by strong predictive performance (PPV = 92.9%, NPV = 90.0%). Logistic Regression also demonstrated robust performance, particularly in terms of PPV. In contrast, SVM yielded markedly lower specificity and predictive values, indicating limited clinical utility in its current configuration.

**Table 2:** Classification performance comparison of combined models

	Sensitivity (%)	Specificity (%)	PPV (%)	NPV (%)
XGBoost	89.7	93.1	92.9	90.0
LR	87.1	93.1	93.1	87.1
RF	80.6	89.7	89.3	81.3
SVM	75.9	32.3	51.2	58.8

*PPV* positive predictive value, *NPV* negative predictive value

**Fig. 3:**
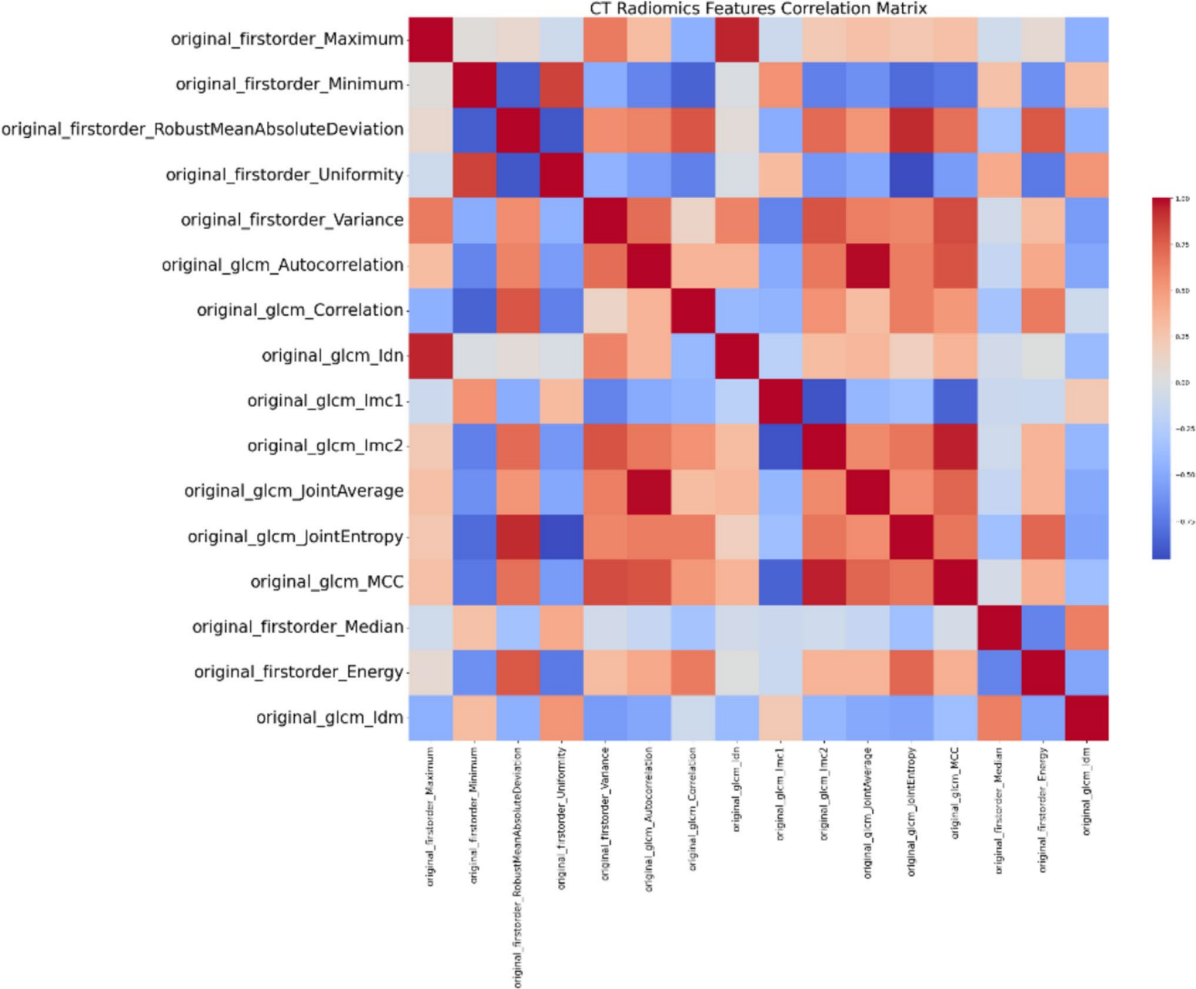
CT radiomics features correlation matrix

**Fig. 4:**
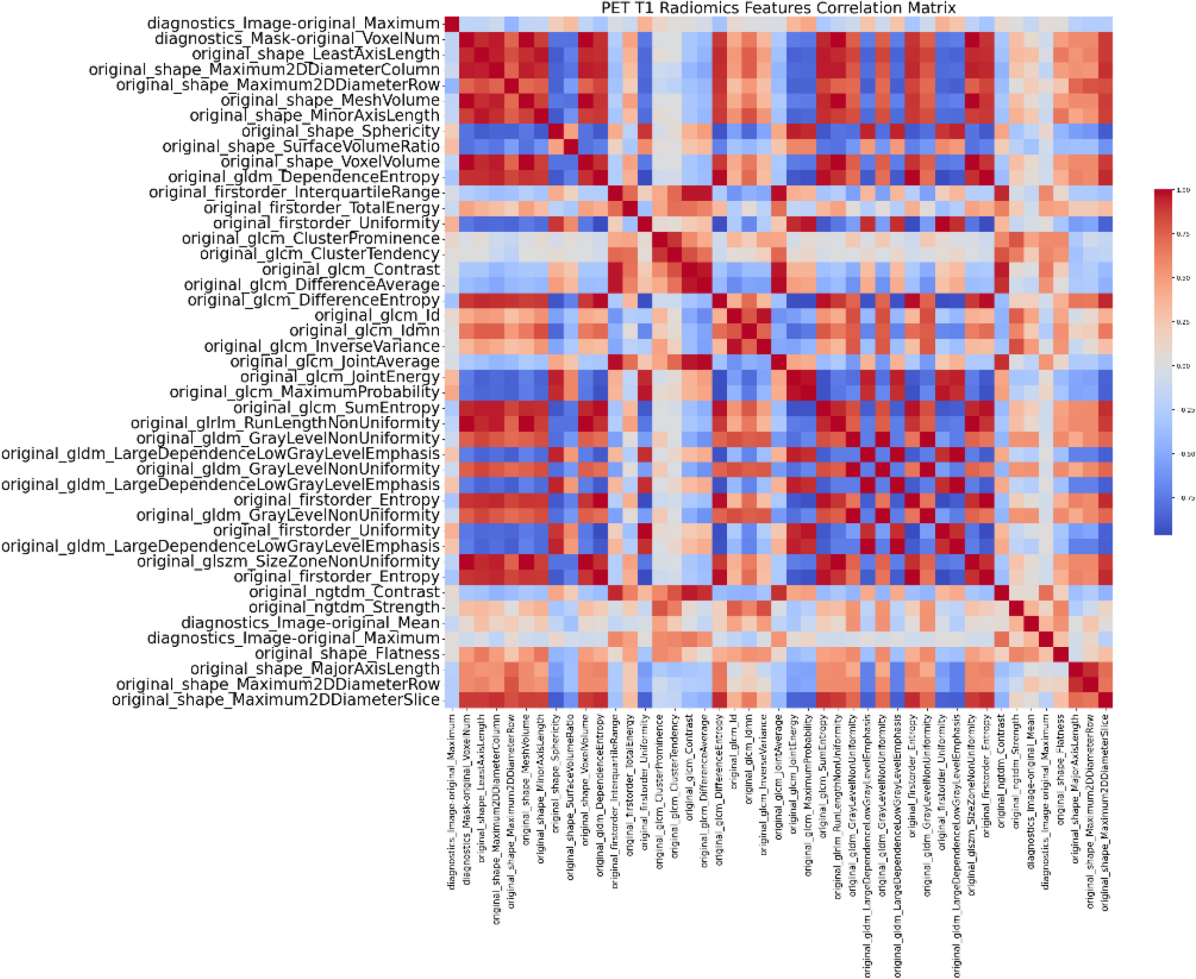
PET T1 radiomics features correlation matrix

**Fig. 5:**
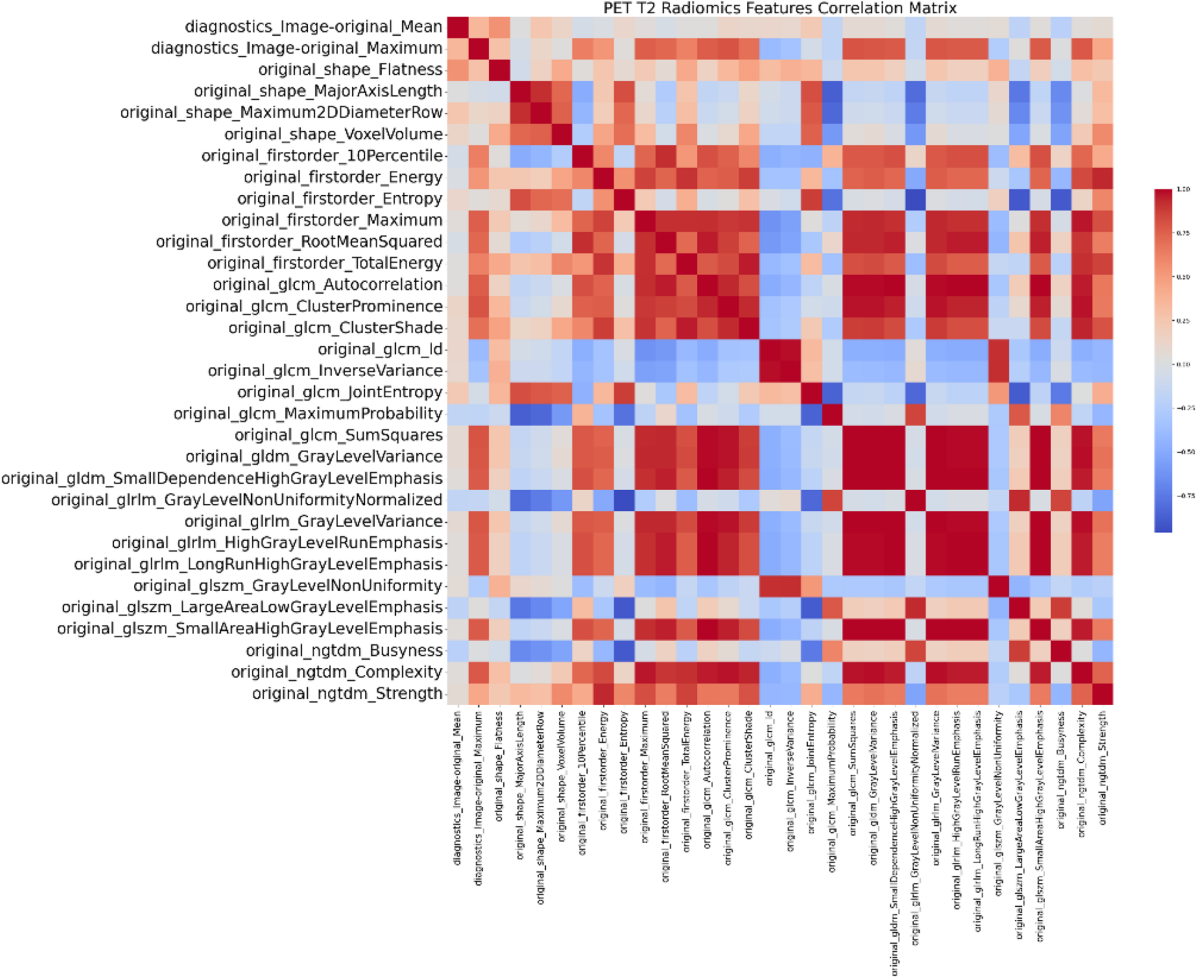
PET T2 radiomics features correlation matrix

Additionally, three distinct heatmaps were generated to illustrate the correlation matrices of various radiomic features as Figs. 3, 4, 5 below. The first heatmap represents the correlation matrix of 16 radiomic features extracted from CT images. The second heatmap, corresponding to PET T1, includes 41 radiomic features, while the third heatmap, corresponding to PET T2, encompasses 32 radiomic features. These heatmaps elucidate the relationships among the features, highlighting potential redundancies and interdependencies.

### Decision curve analysis

**Fig. 6:**
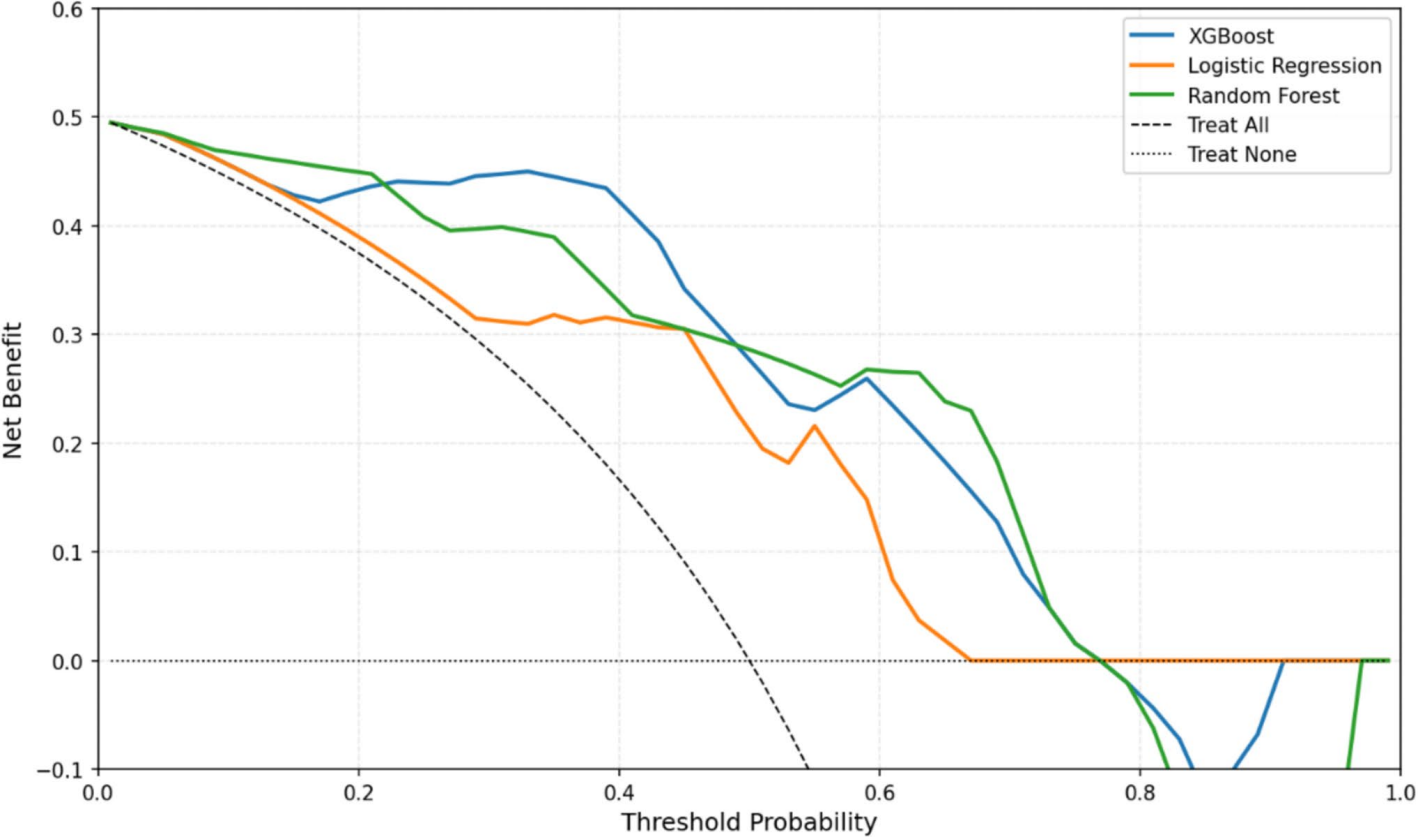
Decision curve analysis (XGBoost vs. logistic regression vs. random forest)

Decision curve analysis revealed distinct net benefit patterns across threshold probabilities for the three models (Fig. 6). The XGBoost model achieved the highest net benefit at low thresholds (0.05–0.30), maintaining stable performance without significant fluctuations. In contrast, logistic regression dominated the mid-threshold range (0.30–0.55), with a peak net benefit of 0.42 at 0.40, but exhibited declining utility beyond 0.55, reaching negative net benefits at thresholds > 0.70. Random forest demonstrated marked variability, with transient peaks at 0.25 (net benefit: 0.38) and 0.50 (0.35), but sharp declines in adjacent ranges (e.g., 0.30–0.45 and 0.55–0.65).

## Discussion

This study highlighted the effectiveness of integrating PET/CT imaging data with machine learning models to predict early chemotherapy responses in breast cancer patients. Among the models evaluated, XGBoost demonstrated the highest predictive capability, achieving an Area AUC value of 0.85. This performance exceeded that of RF (AUC: 0.76), LR (AUC: 0.80), and SVM (AUC: 0.59). The superior performance of the XGBoost model underscores its potential as a powerful tool for early chemotherapy response prediction, enabling more precise and personalized treatment strategies for breast cancer patients.

In this context, XGBoost emerges as a robust solution capable of addressing these limitations. XGBoost is designed to handle large-scale datasets with superior computational efficiency and enhanced resistance to overfitting [21, 22]. This algorithm applies gradient boosting principles to iteratively refine prediction accuracy, making it adept at managing complex interactions within high-dimensional data. Therefore, applying XGBoost in predicting breast cancer response should be a key area of focus in current research. Additionally, the application of deep learning models such as the squeeze-and-excitation network (SENet) further enhances the ability to capture significant imaging features from PET/CT data, providing more robust inputs for predictive modeling [23].

By incorporating deep learning-derived features extracted from PET/CT images using the SENet model, the predictive performance of all machine learning models markedly improved. The AUC values for XGBoost, LR, RF, and SVM increased to 0.92, 0.90, 0.88, and 0.61, respectively. These results underscore the significant value of integrating deep learning-extracted features with traditional radiomic features, enhancing the precision and reliability of treatment planning. While the SVM model continued to underperform compared to other models, the substantial improvement in the XGBoost, LR, and RF models highlights the potential of deep learning-derived features to augment the predictive power of conventional radiomic analysis. The statistically validated superiority of XGBoost underscores its capability to leverage both radiomic and deep learning-derived features, whereas SVM’s limitations in handling nonlinear interactions may explain its suboptimal performance.

The superior performance of the XGBoost model can be largely attributed to its sophisticated algorithmic design, which effectively handles complex interactions among features and exhibits robustness in managing high-dimensional datasets [24]. This model’s predictive accuracy is significantly bolstered by its capacity to incorporate and analyze a diverse array of radiomic features. Specifically, the interquartile range of the GTV at the pre-treatment stage (T1) and the difference in mean standardized uptake value (SUVmean) between the pre-treatment stage (T1) and the post-first chemotherapy cycle (T2) emerged as critical predictors. These features likely capture essential variations in tumor metabolism and morphological changes induced by chemotherapy, which are pivotal for assessing treatment efficacy. The detailed and dynamic nature of these predictors contributes to the enhanced reliability and accuracy of the XGBoost model. Furthermore, the model’s ability to integrate semi-quantitative PET parameters and clinical features underscores its comprehensive approach to predicting chemotherapy response, thereby reinforcing its potential as a valuable tool in the realm of personalized cancer treatment.

A potential critique of our methodology lies in its deviation from the conventional use of DL models, which are typically employed to predict outcomes directly without explicit feature extraction [25, 26]. In contrast, our approach utilized DL primarily for feature extraction, effectively reframing it into a handcrafted feature model. While this approach may seem unconventional, it enables a more interpretable integration of deep learning-derived features with machine learning models, allowing for a clearer understanding of which features are most predictive of chemotherapy response.

Our results indicate that deep features do not merely refine existing radiomics but offer complementary information. This is supported by the observed improvement in AUC when deep features were added to the radiomic feature set, as shown in Fig. 2 and Table 1. If deep features were simply redundant with radiomics, such an improvement would likely not occur due to feature redundancy or overfitting. Instead, the enhanced performance suggests that DL models may capture subtle imaging features that radiomics descriptors are not designed to extract. Moreover, prior investigations have corroborated our findings, demonstrating that the integration of handcrafted radiomic features with deep learning-derived representations yields superior prognostic performance compared to either feature set used in isolation (3). By combining DL-derived features with traditional radiomic analysis, our methodology offers a flexible and scalable framework, capable of adapting to future advancements in medical imaging and DL technologies.

In addition to overall AUC performance, we further evaluated each model’s sensitivity, specificity, PPV, and NPV at clinically relevant thresholds to assess their practical utility in identifying non-responders (NR) after the first chemotherapy cycle. This subgroup is of particular clinical interest, as early identification can prompt timely treatment modifications. As summarized in Table 2, the XGBoost model achieved the most balanced performance, with a sensitivity of 89.7%, specificity of 93.1%, PPV of 92.9%, and NPV of 90.0%. These metrics indicate strong capability in correctly identifying both true responders and non-responders, which is essential for minimizing overtreatment and ensuring early intervention for high-risk patients.

Notably, for DCA curve, although both the XGBoost and Random Forest models outperformed the “Treat All” and “Treat None” strategies across a substantial portion of the threshold probability range, demonstrating their potential value in clinical decision-making. These threshold ranges correspond to practical clinical scenarios where early identification of non-responders is critical. For instance, at a decision threshold of 0.30 using the XGBoost model, approximately 45% of patients could be correctly re-assigned to a more appropriate treatment strategy, with a meaningful net benefit over both “treat all” and “treat none” policies. Similarly, Logistic Regression at threshold 0.40 enables accurate reallocation in approximately 40% of patients, supporting its clinical relevance in mid-risk stratification.

The observed fluctuations in Random Forest, particularly the drop in net benefit beyond 0.55, highlight the model’s instability in high-risk prediction and caution against its use in decision-making at these thresholds. Notably, all three models demonstrate near-zero or negative net benefit at thresholds > 0.75, suggesting limited clinical utility in extreme risk categories where false positives or false negatives may dominate. These findings underscore the importance of selecting clinically meaningful threshold probabilities when applying predictive models in practice. XGBoost and LR, in particular, offer robust utility in early to intermediate decision ranges, potentially enabling timely modification of therapeutic strategies and improving patient outcomes. Nonetheless, the limited sample size (*N* = 60) likely contributes to curve instability, particularly at higher thresholds. This highlights the need for larger, multicenter datasets to refine model calibration and validate net benefit consistency across risk strata.

Our findings are consistent with previous research that underscores the potential of machine learning models in predicting cancer treatment outcomes. Several studies have illustrated how the integration of imaging data with machine learning algorithms can enhance predictive accuracy, thereby improving treatment planning and patient management. For instance, studies by Cai et al. and Zhang et al. have demonstrated that radiomic features extracted from imaging data can serve as valuable predictors of treatment response in various cancers [9, 27].

The strength of our approach lies in the comprehensive integration of PET/CT imaging data with machine learning models, which allows for a more nuanced and detailed analysis of tumor characteristics and treatment responses. This methodology leverages the strengths of both imaging and computational analysis, facilitating a deeper understanding of the factors that influence chemotherapy efficacy. However, several potential limitations must be acknowledged. The retrospective design of our study may introduce inherent biases, as the data were collected and analyzed after the fact, which can affect the interpretation of causality and outcome relationships. Additionally, a large sample size would enhance generalizability of our findings, providing a more robust validation of the model’s predictive power. The inclusion of a diverse patient population and multi-institutional data could further enhance the reliability and applicability of the results. Future research should focus on prospective studies with larger sample sizes to validate our findings and address these limitations. Moreover, incorporating additional predictive variables, such as genomic data and more comprehensive clinical features, could potentially enhance the models’ accuracy and applicability, paving the way for more personalized and effective cancer treatment strategies.

The ability to predict chemotherapy responses early in the treatment process holds substantial clinical implications. Early identification of patients unlikely to respond to standard chemotherapy regimens enables clinicians to adjust treatment plans promptly, potentially opting for alternative therapies such as radiation or hormone therapy. This personalized approach optimizes treatment efficacy and minimizes unnecessary exposure to ineffective treatments, thereby improving patient outcomes and reducing healthcare costs [28, 29]. Early prediction allows for a more tailored therapeutic strategy, ensuring that each patient receives the most appropriate and effective treatment based on their specific tumor characteristics and predicted response.

One important limitation of this study is the use of imaging and clinical data from a single-center retrospective dataset (QIN-Breast), sourced from The cancer imaging archive (TCIA). Although this publicly available dataset provides a standardized and well-curated imaging resource, the lack of an independent external validation cohort restricts the generalizability of our findings. Variations in imaging acquisition protocols, scanner characteristics, and patient demographics across institutions may influence model performance in real-world settings. As such, while the present results are promising, they should be interpreted within the context of this dataset constraint. The dataset also lacks tumor biology data, such as hormone receptor status, HER2 expression, and molecular subtype. These biological characteristics are known to influence treatment response and prognosis in breast cancer, and their absence may limit the interpretability and generalizability of our predictive models. Future studies are being planned to validate the proposed deep radiomic models using larger, multi-institutional cohorts that encompass broader imaging heterogeneity and population diversity. These efforts will be essential to establish the robustness and clinical applicability of the model prior to prospective clinical deployment.

Future research should focus on prospective studies to validate the predictive models developed in this study. Additionally, incorporating more predictive variables, such as genomic data and additional radiomic features, could enhance the models’ accuracy and applicability. Testing these models in real-world clinical settings will also be crucial to assess their practical utility and refine them based on clinical feedback.

## Conclusion

In conclusion, our study demonstrated that integrating PET/CT imaging data with machine learning models, including XGBoost, SVM, LR, and RF model, effectively predicts early chemotherapy responses in breast cancer patients. The XGBoost model showed superior predictive capability, facilitating personalized treatment strategies. Early identification of non-responders allows for timely interventional therapy, enhancing treatment efficacy and avoiding unnecessary continuation of ineffective treatments. This approach underscores the potential of advanced machine learning models in improving patient outcomes and optimizing cancer treatment management.

## Data Availability

The datasets generated and/or analysed during the current study are available from the corresponding author upon reasonable request.

## References

[1] Sung H, et al. Global cancer statistics 2020: GLOBOCAN estimates of incidence and mortality worldwide for 36 cancers in 185 countries. CA Cancer J Clin. 2021;71(3):209–49. 33538338 10.3322/caac.21660

[2] Łukasiewicz S, et al. Breast cancer—epidemiology, risk factors, classification, prognostic markers, and current treatment strategies—an updated review. Cancers. 2021;13(17):4287. 34503097 10.3390/cancers13174287 PMC8428369

[3] Fang S, et al. A real-world clinicopathological model for predicting pathological complete response to neoadjuvant chemotherapy in breast cancer. Front Oncol. 2024;14:1323226. 38420013 10.3389/fonc.2024.1323226 PMC10899694

[4] Hadebe B, et al. The role of PET/CT in breast cancer. Diagnostics. 2023;13(4): 597. 36832085 10.3390/diagnostics13040597 PMC9955497

[5] Li T, et al. Applications of FAPI PET/CT in the diagnosis and treatment of breast and the most common gynecologic malignancies: a literature review. Front Oncol. 2024;14:1358070. 38505595 10.3389/fonc.2024.1358070 PMC10949888

[6] Iqbal MJ, et al. Clinical applications of artificial intelligence and machine learning in cancer diagnosis: looking into the future. Cancer Cell Int. 2021;21(1):270. 34020642 10.1186/s12935-021-01981-1 PMC8139146

[7] Shah SM, et al. Leveraging machine and deep learning algorithms for hERG blocker prediction. IEEE Access. 2025;13:810.

[8] Ariga K. Responsive materials nanoarchitectonics at interfaces. Respons Mater. 2024;2(2): e20240011.

[9] Zhang B, Shi H, Wang H. Machine learning and AI in cancer prognosis, prediction, and treatment selection: a critical approach. J Multidisc Healthc. 2023;2023:1779–91. 10.2147/JMDH.S410301 PMC10312208 37398894

[10] Rasool A, et al. Improved machine learning-based predictive models for breast cancer diagnosis. Int J Environ Res Public Health. 2022;19(6):3211. 35328897 10.3390/ijerph19063211 PMC8949437

[11] Zhang Y, et al. PET radiomics in lung cancer: advances and translational challenges. EJNMMI Phys. 2024;11(1):81. 39361110 10.1186/s40658-024-00685-5 PMC11450131

[12] Zhou Y, et al. Integrating 18F-FDG PET/CT radiomics and body composition for enhanced prognostic assessment in patients with esophageal cancer. BMC Cancer. 2024;24(1):1402. 39543534 10.1186/s12885-024-13157-x PMC11566154

[13] Menon N, et al. Performance of radiomics-based artificial intelligence systems in the diagnosis and prediction of treatment response and survival in esophageal cancer: a systematic review and meta-analysis of diagnostic accuracy. Dis Esophagus. 2023;36(6):doad034. 37236811 10.1093/dote/doad034 PMC10789236

[14] Sim Y, et al. Multiparametric MRI–based radiomics model for predicting human papillomavirus status in oropharyngeal squamous cell carcinoma: optimization using oversampling and machine learning techniques. Eur Radiol. 2024;34(5):3102–12. 37848774 10.1007/s00330-023-10338-3

[15] Lohmann P, et al. Radiomics in radiation oncology—basics, methods, and limitations. Strahlenther Onkol. 2020;196(10):848–55. 32647917 10.1007/s00066-020-01663-3 PMC7498498

[16] Mali SA, et al. Making radiomics more reproducible across scanner and imaging protocol variations: a review of harmonization methods. J Personal Med. 2021;11(9):842. 10.3390/jpm11090842 PMC8472571 34575619

[17] Liu Y, et al. Multimodality deep learning radiomics predicts pathological response after neoadjuvant chemoradiotherapy for esophageal squamous cell carcinoma. Insights Imaging. 2024;15(1): 277. 39546168 10.1186/s13244-024-01851-0 PMC11568088

[18] Wei W, et al. Deep learning radiomics for prediction of axillary lymph node metastasis in patients with clinical stage T1–2 breast cancer. Quant Imaging Med Surg. 2023;13(8):4995. 37581073 10.21037/qims-22-1257 PMC10423344

[19] Li X, et al. Data from QIN-breast. 2016. p. 5.

[20] Wahl RL, et al. From RECIST to PERCIST: evolving considerations for PET response criteria in solid tumors. J Nucl Med. 2009;50(Suppl 1):122S-150S. 19403881 10.2967/jnumed.108.057307 PMC2755245

[21] Zhang X, et al. Predicting missing values in medical data via XGBoost regression. J Healthc Inform Res. 2020;4:383–94. 33283143 10.1007/s41666-020-00077-1 PMC7709926

[22] Xu Y, et al. Predicting patients’ satisfaction with doctors in online medical communities: an approach based on XGBoost algorithm. J Organ End User Comput. 2022;34(4):1–17.

[23] Hu J, Shen L, Sun G. Squeeze-and-excitation networks. In: Proceedings of the IEEE conference on computer vision and pattern recognition. 2018.

[24] Ma B, et al. XGBLC: an improved survival prediction model based on XGBoost. Bioinformatics. 2022;38(2):410–8. 34586380 10.1093/bioinformatics/btab675

[25] Singh AV, et al. Investigating tattoo pigments composition with UV-Vis and FT-IR spectroscopy supported by chemometric modelling. Curr Anal Chem. 2024;1–12:2024.

[26] Chandrasekar V, et al. Quantitative prediction of toxicological points of departure using two-stage machine learning models: a new approach methodology (NAM) for chemical risk assessment. J Hazard Mater. 2025;487:137071. 39808958 10.1016/j.jhazmat.2024.137071

[27] Cai J, et al. A radiomics model for predicting the response to bevacizumab in brain necrosis after radiotherapy. Clin Cancer Res. 2020;26(20):5438–47. 32727886 10.1158/1078-0432.CCR-20-1264

[28] Romeo V, et al. Assessment and prediction of response to neoadjuvant chemotherapy in breast cancer: a comparison of imaging modalities and future perspectives. Cancers. 2021;13(14):3521. 34298733 10.3390/cancers13143521 PMC8303777

[29] Liang X, et al. Early prediction of pathological complete response to neoadjuvant chemotherapy combining DCE-MRI and apparent diffusion coefficient values in breast cancer. BMC Cancer. 2022;22(1):1250. 36460972 10.1186/s12885-022-10315-x PMC9716688

